# Composition and diverse differences of intestinal microbiota in ulcerative colitis patients

**DOI:** 10.3389/fcimb.2022.953962

**Published:** 2022-08-30

**Authors:** Siying Zhu, Muzhou Han, Simao Liu, Liqiaona Fan, Haiyun Shi, Peng Li

**Affiliations:** Department of Gastroenterology, Beijing Friendship Hospital, Capital Medical University; National Clinical Research Center for Digestive Diseases; Beijing Digestive Disease Center, Beijing, China

**Keywords:** ulcerative colitis, intestinal microbiota, bacterial, composition, diversity

## Abstract

**Objective:**

To explore the composition of the intestinal microbiota in ulcerative colitis (UC) patients and to identify differences in the microbiota between patients with active disease and those in remission.

**Methods:**

Between September 2020 and June 2021, we enrolled into our study, and collected stool samples from, patients with active UC or in remission and healthy control subjects. The diagnosis of UC was based on clinical, endoscopic, radiological, and histological findings. The composition of the intestinal microbiota was determined by sequencing of the 16S rRNA V3–V4 region and by bioinformatic methods. The functional composition of the intestinal microbiota was predicted using PICRUSt 2 (Phylogenetic Investigation of Communities by Reconstruction of Unobserved States) software.

**Results:**

We found that the intestinal flora was significantly less rich and diverse in UC patients than in healthy control subjects. Beta diversity analysis revealed notable differences in the intestinal flora compositions among the three groups, but there was no statistical difference in alpha diversity between UC patients with active disease and those in remission. At the phylum level, the relative abundances of *Proteobacteria* and *Patescibacteria* were significantly higher, and the relative abundances of *Desulfobacterota* and *Verrucomicrobiota* were lower, in UC patients with active disease than in the healthy control group. Higher levels of potential pathogens and lower levels of butyrate-producing bacteria were also detected in UC patients with active disease. Linear discriminant analysis Effect Size (LefSe) revealed that 71 bacterial taxa could serve as biomarkers, with 26 biomarkers at the genus level. In addition, network analysis showed that there was a positive correlation between *Roseburia* and *Lachnospira.* Functional predictions indicated that gene functions involving the metabolism of some substances, such as methane, lipopolysaccharide, geraniol, and ansamycins, were significantly different among the three groups.

**Conclusion:**

The richness and diversity of the intestinal microbiota differed significantly among the three groups. Richness describes the state of being rich in number of intestinal bacteria, whereas diversity is the number of different species of intestinal bacteria. Different bacterial taxa could be used as biomarkers, expanding our understanding of the relationship between the intestinal microbiota microenvironment and UC in the future.

## Introduction

Ulcerative colitis (UC) and Crohn’s disease (CD) are clinical subtypes of inflammatory bowel disease (IBD) and are chronic, relapsing immune-related disorders of the gastrointestinal tract of unknown etiology. Individuals with UC and CD often present with symptoms such as persistent diarrhea, abdominal pain, and hematochezia (Malik et al., 2018). In 2017, it was estimated that over 6.8 million people worldwide suffered from IBD (GBD 2017 Inflammatory Bowel Disease Collaborators, 2020). The total number of cases of IBD is likely to continue to increase, and IBD is expected to become an enormous public health burden in the next decade (Ng et al., 2017). Using all means possible to identify the cause(s) of IBD and intervene appropriately will be the top priority of future IBD medical care.

Currently, it is widely believed that intestinal flora, which comprises over 100 trillion bacteria, as well as myriads of fungi, viruses (Saeedi et al., 2020), and protozoa, play a significant role in the pathogenesis of IBD (Sartor, 2008). Bacteria could influence intestinal inflammation through interaction with the immune system (Sabino et al., 2016), for example causing up-regulation of anti-inflammatory cytokines or down-regulation of pro-inflammatory cytokines (Kostic et al., 2014). It is known that the contribution of genetic factors to disease etiology is greater for CD than for UC, and so the impact of intestinal dysbiosis on UC may be worthy of further exploration (Tremelling et al., 2007). Previous studies have shown that, compared with healthy people, the gut of UC patients contains fewer *Firmicutes* organisms and more *Bacteroidetes* organisms and facultative anaerobes (Hansen et al., 2010). In addition, the relative abundance of *Enterobacteriaceae*, in particular *Escherichia coli* and *Shigella* species, is increased by the aggravation of intestinal inflammation, whereas the opposite is true of *Lachnospiraceae* and *Ruminococcaceae* (He et al., 2021). Increased understanding of the changes in the microbiota associated with UC has led to the development of microbiota-related therapeutic strategies, such as the widespread use of probiotic preparations and fecal microbiota transplantation. However, more research into the specific changes that occur in the enteric microbiota and the possible pathogenic mechanism is needed to clarify the relationship between the gut microbiota and the clinical manifestation of UC.

Using 16S rRNA gene amplicon sequencing, in this study we explored the diversity and compositional differences of gut bacterial communities in UC patients with active disease, UC patients in remission, and healthy individuals, which expanded our understanding of intestinal bacterial ecology in UC.

## Materials and methods

### Patients and specimens

For this study, 40 UC patients ranging in age from 18 to 75 years were enrolled at Beijing Friendship Hospital Affiliated to Capital Medical University from September 2020 to June 2021. To be eligible for the study, UC patients were required to have had a clinical disease for more than 3 months. The diagnosis of UC was based on clinical, endoscopic, radiological, and histological findings in accordance with consensus on the diagnosis and treatment of IBD (Nakase et al., 2021). In addition, the level of disease activity was determined by the Mayo score/Disease Activity Index, and UC patients were classified as having an active disease or being in remission (Schroeder et al., 1987). A total of 20 sex-matched healthy control subjects were also enrolled, that is, healthy volunteers unrelated to the patients in this study. None of the participants were taking antibiotics, antacids, non-steroidal anti-inflammatory drugs, antiplatelet drugs, anticoagulants, or glucocorticoids, and participants had a history of colectomy in the 3 months prior to stool sample collection. Fecal specimens collected from patients and healthy control subjects were immediately added to buffer solution and cryopreserved at –20°C until sequencing.

All specimen studies were approved by the Medical Ethical Committee of the Beijing Friendship Hospital Affiliated to the Capital University of Medical Sciences (number 2019-P2-233-01) and conducted in accordance with the principles expressed in the Declaration of Helsinki. Written informed consent was obtained from all patients and healthy individuals participating in this study.

### DNA extraction and 16S ribosomal RNA gene sequencing

DNA was extracted from fecal specimens (approximately 150 mg per stool sample) using a QIAamp DNA Stool Mini Kit (Qiagen, Hilden, Germany) in accordance with the manufacturer’s protocols. The V3–V4 region of the bacterial 16S rRNA gene was amplified by PCR (94 °C for 5 min, followed by 30 cycles at 94 °C for 30 s, 50 °C for 30 s, 72°C for 60 s, then 72 °C for 7 min) with the forward primer 338F (5′-ACTCCTACGGGAGGCAGCA-3′) and the reverse primer 806R (5′-GGACTACHVGGGTWTCTAAT-3′). PCR products were electrophoresed on a 1% agarose gel to identify the size of the amplified target band and purified using the Agencourt AMPure XP Nucleic Acid Purification Kit (Beijing, China). Sequencing was performed using the MiSeqPE300 platform from Illuminate.

### Bioinformatics analyses

The pyrosequencing data were processed using Quantitative Insights Into Microbial Ecology (QIIME) v. 1.8.0 (Nakase et al., 2016) and VSEARCH (v. 2.7.1) software (Rognes et al., 2016). Operational taxonomic units (OTUs) with a threshold of 97% sequence similarity were clustered using UPARSE (version 7.1) (Edgar, 2013). The taxonomy of each OTU representative sequence was analyzed using the Ribosomal Database Project Classifier (Cole et al., 2009; Sabino et al., 2016) and compared against the SILVA database (release 138) (Quast et al., 2013) using a confidence threshold of 0.7. FastTree (Price et al., 2009) software was used to construct a phylogenetic tree, and Python was applied to visualize their abundance and evolutionary relationship. The microbial alpha and beta diversities were calculated in QIIME 1.8.0 and visualized in the R environment. Meanwhile, hierarchical clustering analysis was carried out according to the unweighted UniFrac distance, and a dendrogram was constructed using the unweighted pair group method with arithmetic mean (UPGMA).

### Statistical analysis

MetaStats, the Kruskal–Wallis test, analysis of molecular variance, the Wilcoxon rank-sum test, and analysis of similarities (ANOSIM) were all appropriately used to test the significance of differences in species composition and community structure between the groups. Linear discriminant analysis Effect Size (LEfSe) analysis (Segata et al., 2011) was employed to identify species whose abundance differed significantly between the three groups (i.e. biomarkers). Spearman’s test method and the top 20 genus-level results of absolute abundance in all samples were selected for correlation analysis. The calculated results were plotted after filtering out *p-*values above 0.05 or correlation values | *R *| < 0.6. Phylogenetic Investigation of Communities by Reconstruction of Unobserved States (PICRUSt 2.0) (Langille et al., 2013) was applied to predict metagenome function. All significance thresholds were set at *p*-values < 0.05.

## Results

### Characteristics and microbiota basic analysis of the subjects

Forty patients with UC and 20 healthy control subjects were enrolled in this study. The UC patients comprised 20 with active disease and 20 in remission. The following basic clinical characteristics were recorded: age, sex, duration of disease, and disease type distribution (E1/E2/E3) (**Table 1**). No significant differences in the baseline characteristics were observed between UC patients and healthy control subjects.

**Table 1:** Clinical characteristics of the subjects.

Characteristics	UC patients (n=40)		Healthy controls (n=20)
	Active stage (n=20)	Remission stage (n=20)
Age (years)	46.55±15.04	47.25 ± 16.18	50.65 ± 9.43
Gender (male/female)	12/8	12/8	10/10
Duration (months)	69.85 ± 53.18	73.40 ± 67.34	–
Extent			
Ulcerative proctitis (E1)	6	10	–
Left sided colitis (E2)	3	6	–
Pancolitis (E3)	11	4	–

Data are presented as mean ± standard error of mean.

*p< 0.05 compared with healthy controls group.

From the stool samples from the 40 UC patients and healthy control subjects, we obtained a total of 4,825,757 high-quality sequences from the original MiSeq sequencing data (**Supplementary Figure 1A**). A total of 1325 OTUs were identified using 97% sequence similarity as the taxonomic standard at the species level. The number of unique and common OTUs between groups is depicted in a Venn diagram (**Supplementary Figure 1B**).

The rarefaction curve tended to remain flat, and the Shannon–Wiener curve was saturated, which indicated that the number of sequencing data was reasonable and the data can be considered to comprehensively reflect species richness (**Supplementary Figures 2A, B**). The flattening species accumulation curves indicated that the sample size was sufficient. The rank abundance curve demonstrated that the samples from the healthy control group were more species-rich than samples from UC patients, and the species composition of microbial communities was more uniform in samples from healthy control subjects (**Supplementary Figures 2C, D**).

### Decrease in diversity of intestinal flora in ulcerative colitis patients

**Figure 1:**
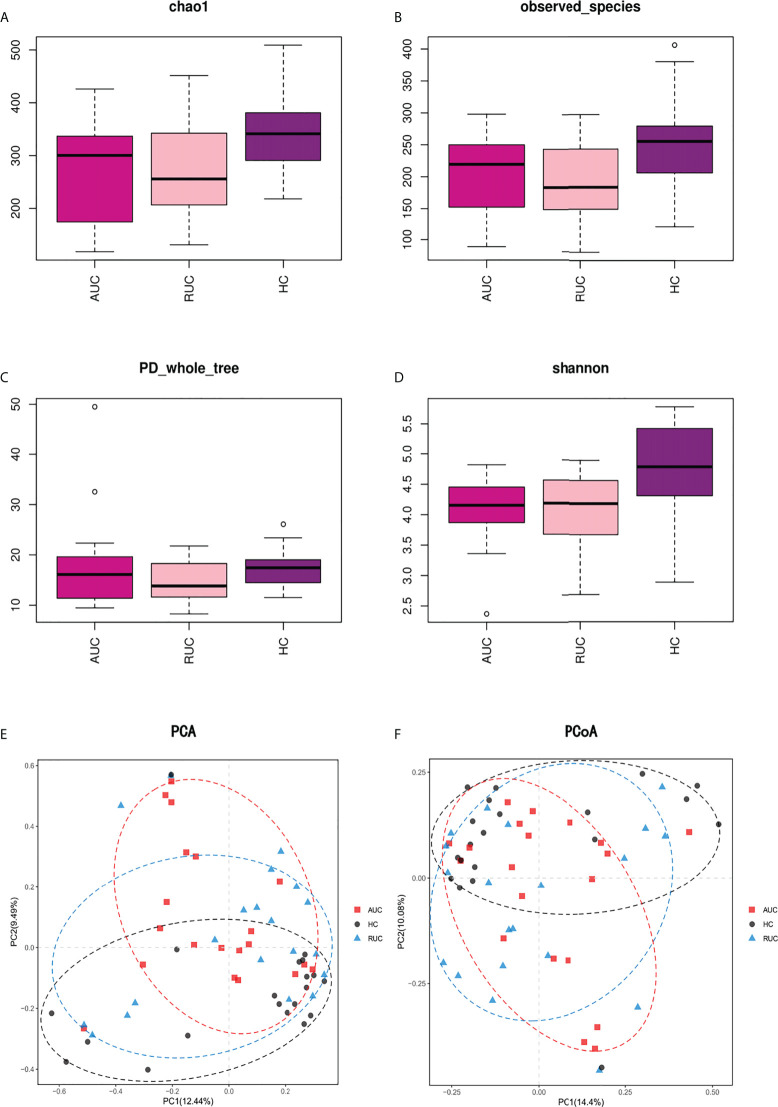
Alpha and beta diversity analysis of the intestinal flora in stool samples from ulcerative colitis patients. **(A–D)**. Results of alpha diversity analysis of intestinal flora as evaluated by Chao 1, observed species, PD Whole Tree, and the Shannon Diversity Index. The diversity of intestinal flora in UC patients in remission was found to be significantly reduced. **(E, F)**. Results of beta diversity analysis. PCA based on OTU leve, and PCoA based on Bray–Curtis distance. Points of different colors or shapes represent different sample groupings, and the scales of the horizontal and vertical axes are relative distances. AUC, UC patients with active disease; RUC, UC patients in remission; and HC, healthy control subjects.

Community richness was evaluated by Chao1. Community diversity was evaluated by the Simpson Diversity Index, the Shannon Diversity Index, and PD Whole Tree (**Figure 1**) using alpha diversity analysis. In addition, Good’s coverage was used to assess sequencing depth, and observed species was used to represent the number of OTUs actually observed with the increase in sequencing depth. Compared with the healthy control subjects [Shannon Diversity Index 4.795 (95% CI 4.33 to 5.42)], the diversity of the intestinal flora among UC patients in remission [Shannon Diversity Index 4.19 (95% CI 3.695 to 4.57)] was significantly lower (*p *< 0.05) (**Figure 1D**). Chao1 and observed species showed that the diversity of the intestinal flora was considerably reduced in UC patients in remission (**Figures 1A, B**).

Euclidean distance and Bray–Curtis distance were determined by principal component analysis (PCA) and principal coordinates analysis (PCoA) respectively, which are types of beta diversity analysis. Both PCA (**Figure 1E**) and PCoA (**Figure 1F**) revealed clear differences in the structure of flora structure between the two UC groups and the control group. Both Adonis (PERMANOVA) analysis (*p* = 0.001) and the ANOSIM test (*r* = 0.0982, *p* = 0.001) indicated significant intergroup differences.

### Taxonomic differences in the gut microbiota of ulcerative colitis patients

**Figure 2:**
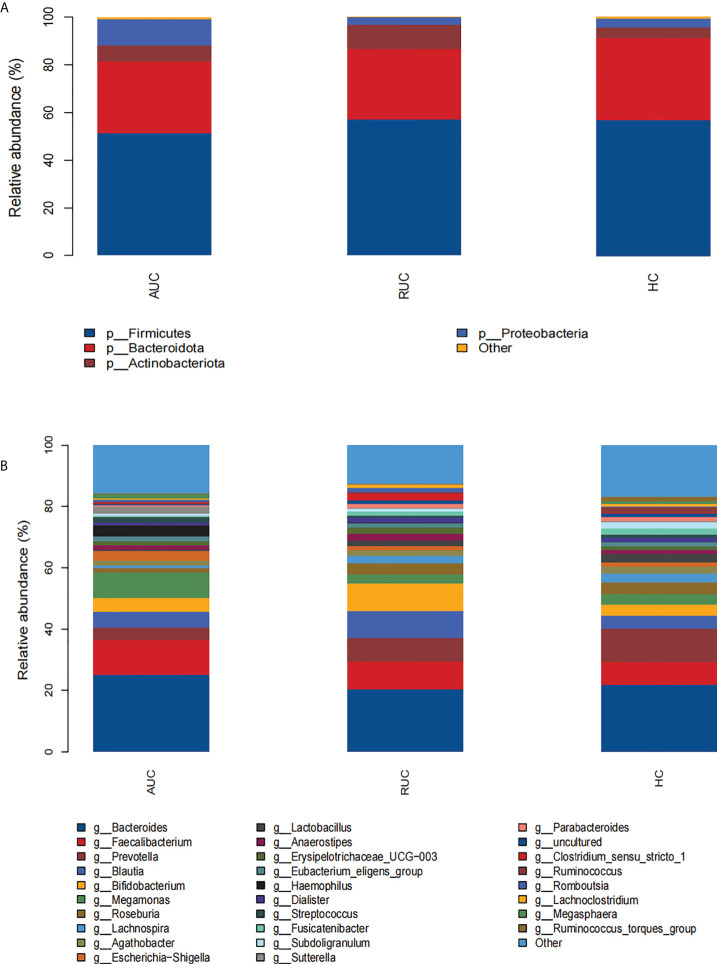
The microbiological compositions at the phylum and genus levels. The stacked bars show the average relative abundances of all phyla and the most common genera and species identified in the three groups. The histograms show species distribution at the phylum **(A)** and genus **(B)** levels in the three groups. AUC, UC patients with active disease; RUC, UC patients in remission; and HC, healthy control subjects.

**Figure 3:**
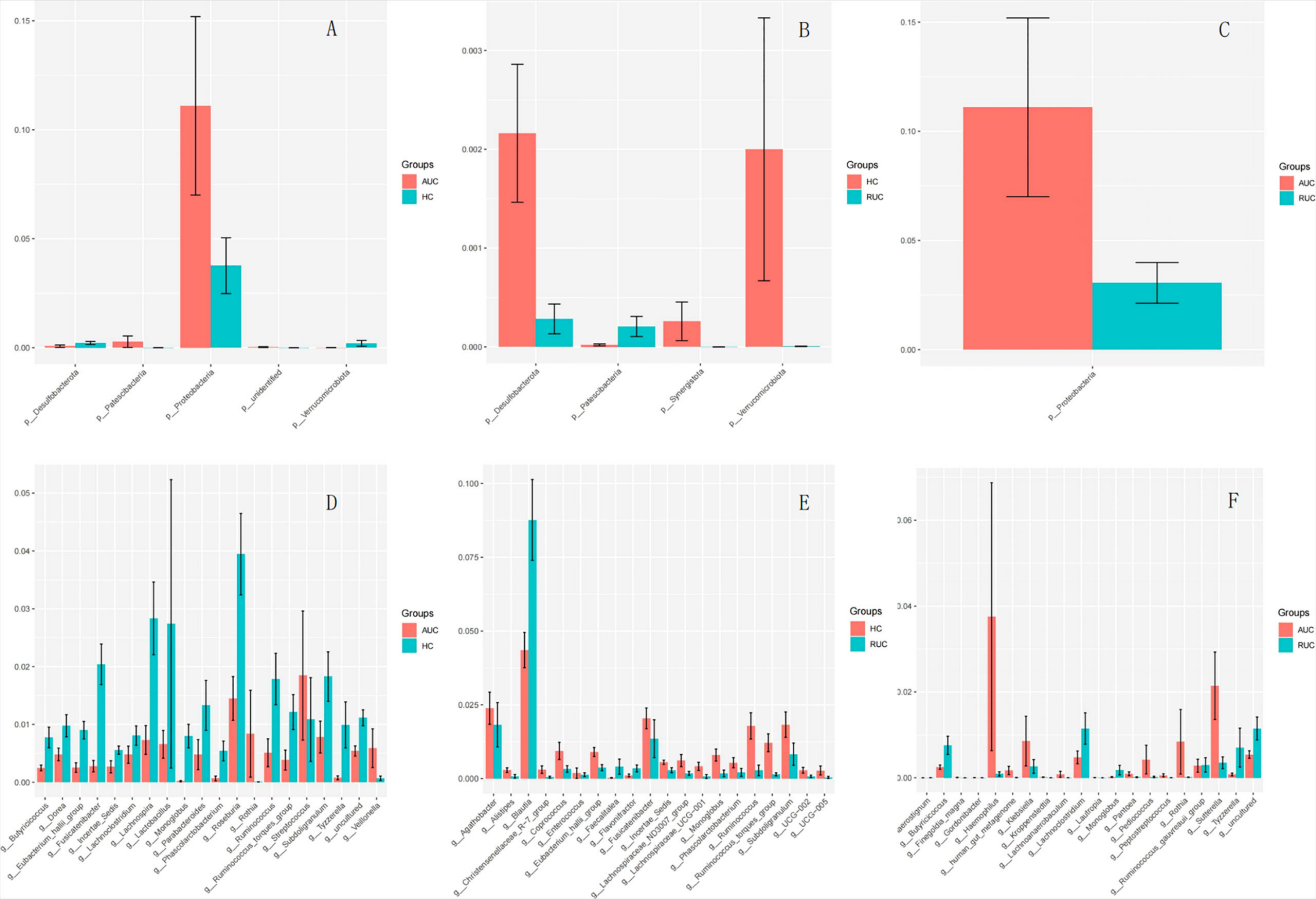
**(A)** Comparison of AUC and HC at the phylum level. **(B)** Comparison of HC and RUC at the phylum level. **(C)** Comparison of AUC and RUC at the phylum level. **(D)** Comparison of AUC and HC at the genus level. **(E)** Comparison of HC and RUC at the genus level. **(F)** Comparison of AUC and RUC at the genus level.

The microbiota composition of the stool samples at the phylum and genus levels were determined by OTU abundance (**Figure 2**). As shown in **Figure 2**, the main phyla in all three groups were *Firmicutes*, *Bacteroidota*, *Actinobacteriota*, and *Proteobacteria*, but their relative abundances were different in each group. In particular, *Firmicutes* was more abundant than *Bacteroidota* and *Actinobacteriota*. The abundance of *Proteobacteria* and *Patescibacteria* was considerably higher in UC patients with active disease than in healthy control subjects (*p *< 0.05), but the opposite was true for *Desulfobacterota* and *Verrucomicrobiota* (**Figure 3A**). The relative abundance of *Patescibacteria* was higher, and the relative abundance of *Desulfobacterota*, *Synergistota*, and *Verrucomicrobiota* was lower, in UC patients in remission than in healthy control subjects (**Figure 3B**). And the abundance of *Proteobacteria* in those with active disease than in those in remission (**Figure 3C**).

At the genus level, the relative abundances of *Rothia*, *Streptococcus*, and *Veillonella* were significantly higher (*p *< 0.05) in UC patients with active disease than in healthy control subjects (**Figure 3D**). In contrast, the relative abundances of *Butyricicoccus*, *Dorea*, *Eubacterium hallii* group, *Fusicatenibacter*, *Incertae sedis*, *Lachnoclostridium*, *Lachnospira*, *Lactobacillus*, *Monoglobus*, *Parabacteroides*, *Phascolarctobacterium*, *Roseburia*, *Ruminococcus*, *Ruminococcus torques* group, *Subdoligranulum*, and *Tyzzerella* were significantly lower in the UC patients with active disease than in the healthy control group (**Figure 3D**). Compared with healthy control subjects, UC patients in remission demonstrated higher relative abundances of *Blautia*, *Faecalitalea*, and *Flavonifractor* and lower relative abundances of *Agathobacter*, *Alistipes*, *Christensenellaceae* R-7 group, *Coprococcus*, *Enterococcus*, *Eubacterium hallii* group, *Fusicatenibacter*, *Incertae sedis*, *Lachnospiraceae* ND3007 group, *Lachnospiraceae* UCG-001, *Monoglobus*, *Phascolarctobacterium*, *Ruminococcus*, *Ruminococcus torques* group, *Subdoligranulum*, UCG-002, and UCG-005 (**Figure 3E**). In addition, there was an increase in the relative abundances of *Finegoldia magna*, *Gordonibacter*, *Haemophilus*, *human gut metagenome*, *Klebsiella*, *Kroppenstedtia*, *Lachnoanaerobaculum*, *Lautropia*, *Pantoea*, *Pediococcus*, *Peptostreptococcus*, *Rothia*, and *Sutterella*. A decrease in the relative abundances of *Anaerostignum*, *Butyricicoccus*, *Lachnoclostridium*, *Monoglobus*, *Ruminococcus gauvreauii group*, and *Tyzzerella* was observed in both groups of UC patients, that is, both those with active disease and those in remission (**Figure 3F**).

**Figure 4:**
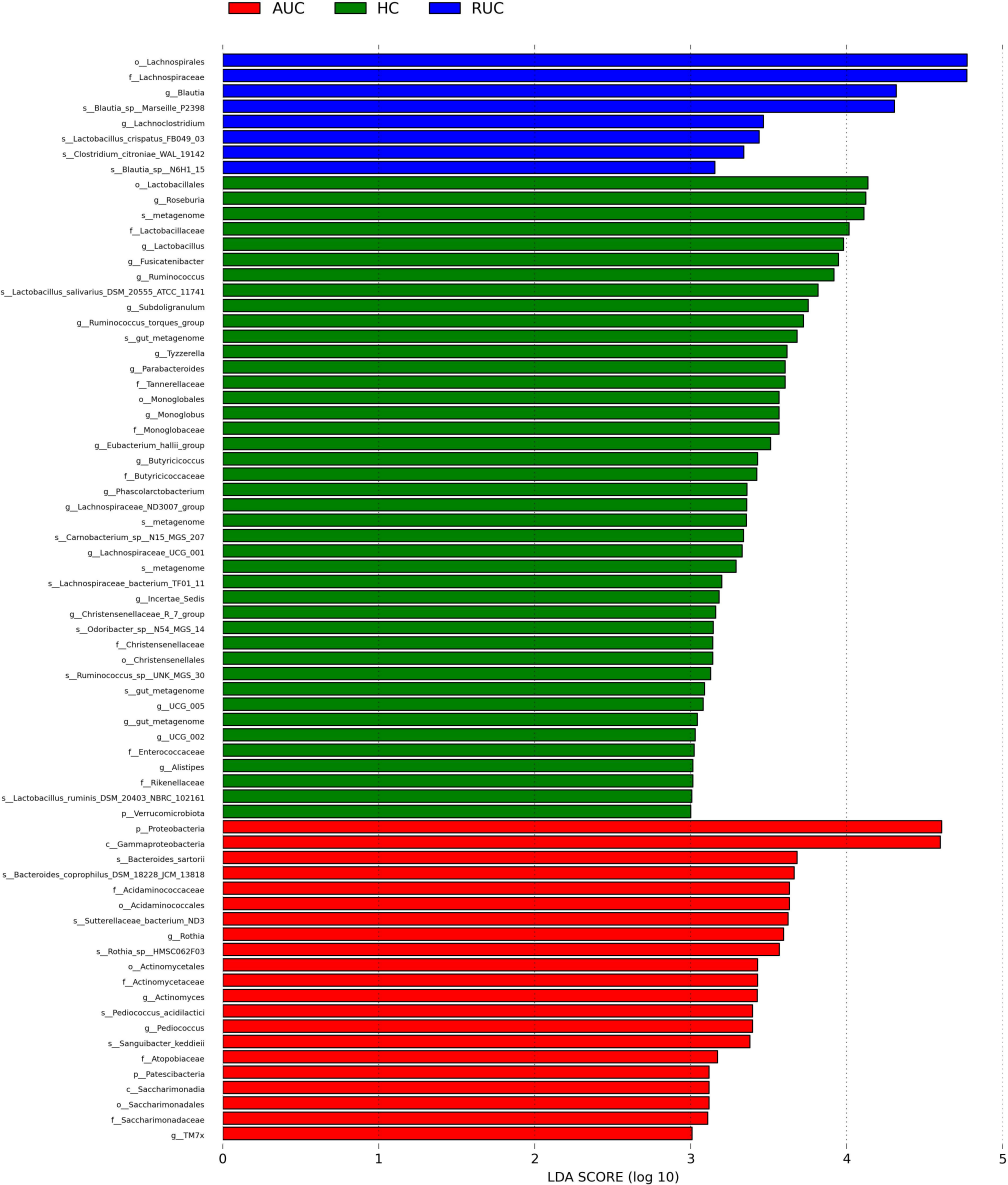
LEfSe analysis of the bacterial communities. LEfSe bar plot of the bacterial communities. AUC, UC patients with active disease; RUC, UC patients in remission; and HC, healthy control subjects.

**Figure 5:**
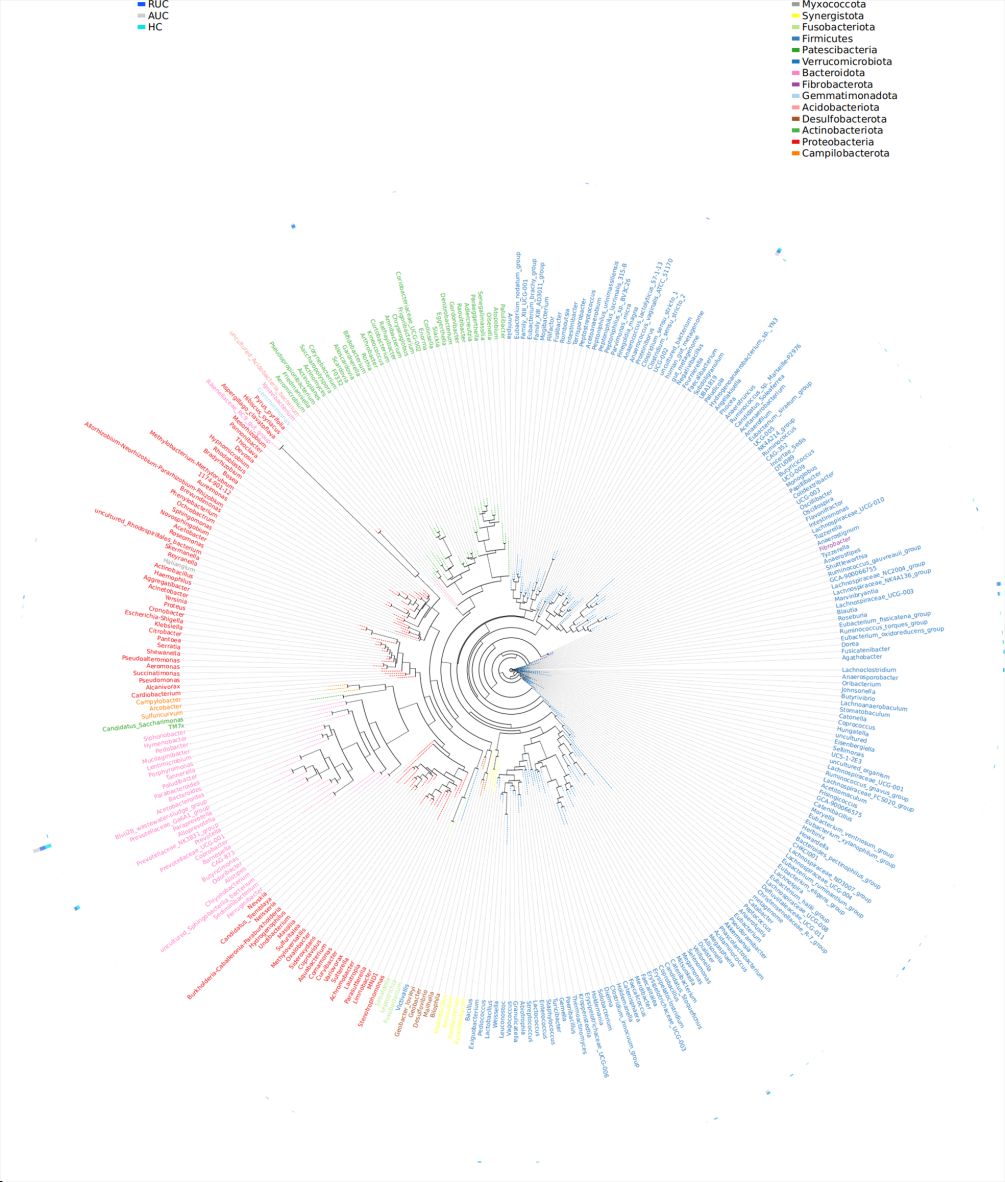
Genus-level evolutionary tree. The first column is the grouping information, and the second column is the phylum level corresponding to the genus level. The representative sequences corresponding to the most abundant OTUs were selected by genus as a unit to build the dendrogram, and the outer circle of the evolutionary tree shows the relative abundance of each genus in each of the different groups. The length of the color blocks represents the relative abundance. AUC, UC patients with active disease; RUC, UC patients in remission; and HC, healthy control subjects.

**Figure 6:**
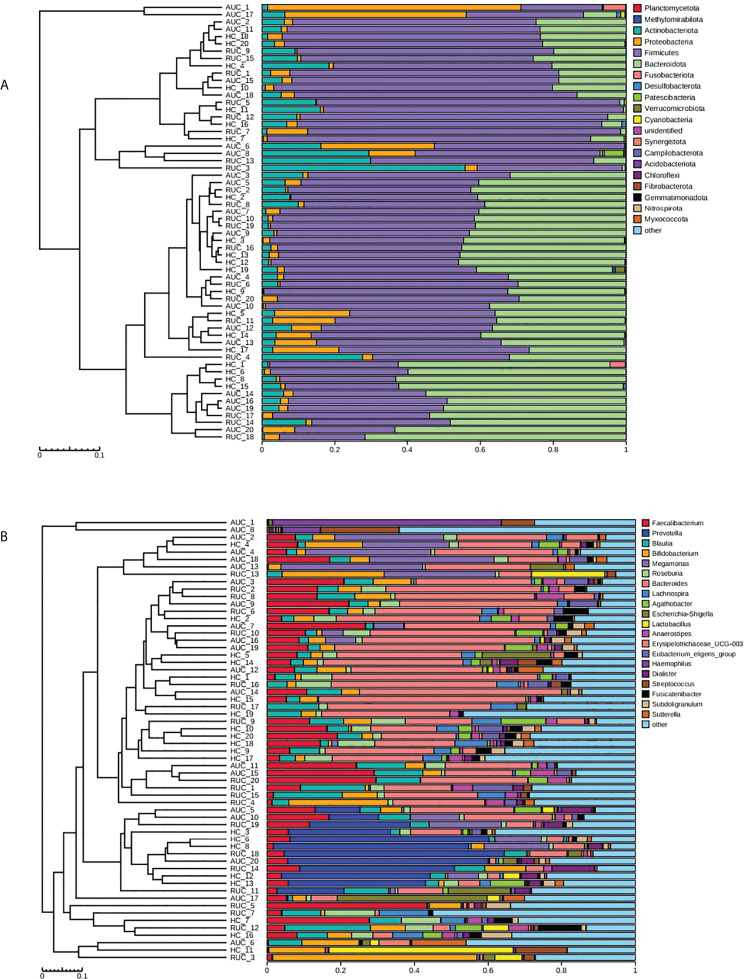
Cluster analysis and dendrogram of microbiota. Left: hierarchical clustering analysis among samples; right: histogram of the community structure of the samples. The abscissa shows the relative abundance of species in the sample, and the ordinate sample name. **(A)** At the phylum level. **(B)** At the genus level. AUC, UC patients with active disease; RUC, UC patients in remission; and HC, healthy control subjects.

LEfSe analysis (a logarithmic LDA score cut-off point of 3.0 and *p*-value < 0.05) revealed remarkable differences among the three groups in 71 bacterial taxa (26 at the genus level) (**Figure 4**). The phylogenetic tree of OTUs at the genus taxonomic level is shown in **Figure 5**. Based on the unweighted UniFrac distance matrix, the UPGMA method was used for cluster analysis and dendrogram, and the clustering results were integrated with the relative abundance of species at the phylum (**Figure 6A**) and genus (**Figure 6B**) levels in each sample.

### The gut microbiota network

Network analysis clearly revealed the interactions among the top 20 gut microbiota in absolute abundance at the genus level. As shown in **Supplementary Figure 3**, there was a positive correlation between *Roseburia* and *Lachnospira* (*p *< 0.05).

### Predicted functional composition of fecal microbial communities

We regarded the microbial functional signatures by implementing PICRUSt 2.0 in the three groups. Differentially abundant metabolic pathways were detected at KEGG level 3 (**Supplementary Figure 4**). Compared with the healthy control subjects, UC patients with active disease exhibited lower expression of genes involved in bacterial chemotaxis, flagellar assembly, pantothenate and coenzyme A biosynthesis, thiamine metabolism, and biosynthesis of ansamycins. However, this group demonstrated higher expression of genes involved in geraniol degradation, fatty acid degradation, ascorbate and aldarate metabolism, valine, leucine and isoleucine degradation, tyrosine metabolism, and propanoate metabolism. UC patients with active disease, compared with those in remission, also demonstrated a higher abundance of geraniol degradation, lipopolysaccharide biosynthesis, toluene degradation, valine, leucine and isoleucine degradation, ubiquinone and other terpenoid-quinone biosynthesis, vitamin B6 metabolism, and lipoic acid metabolism. However, UC patients in remission had a higher abundance of bacterial chemotaxis, methane metabolism, biosynthesis of ansamycins, valine, leucine and isoleucine biosynthesis, lysine biosynthesis, and thiamine metabolism than patients with active disease. Notably, the expression levels of lipopolysaccharide biosynthesis and toluene degradation pathways were significantly lower in UC patients in remission than in healthy control subjects.

## Discussion

In this study, we analyzed the results of high-throughput sequencing of the 16S rRNA V3–V4 variable region of the intestinal flora of UC patients with active disease or in remission and in a healthy population. Significant differences in the diversity and composition of the intestinal flora were identified among the three groups. In particular, alpha diversity in the gut microbiome, evaluated by the observed species, Chao1, and the Shannon Diversity Index, which was significantly lower in UC patients than in healthy control subjects, reflecting the fact that the richness and evenness (i.e. relative density) of bacteria were lower in UC patients than in control subjects. Among UC patients, alpha diversity was lower in those with active disease than in those in remission, but the difference was not statistically significant. Similarly, beta diversity of gut microbiota significantly differed among the three groups. However, the dendrogram based on unweighted UniFrac distance using UPGMA method clustering revealed no clear differences among the three groups. Our findings are analogous to those of previous studies (Ma et al., 2018; Xu et al., 2021). Earlier studies revealed that a reduction in the diversity of fecal microbiota is the most consistent indicator of IBD, and the microbiome of UC patients is more similar to that of healthy control subjects than to that of individuals with CD (Sankarasubramanian et al., 2020).

At the phylum level, the profile of gut microbiota appeared to be dominated by *Firmicutes*, *Bacteroidetes*, and *Proteobacteria*, which is consistent with the findings of previous studies (Forbes et al., 2016; Rehman et al., 2016), confirming the reliability of our samples and analytical methods. The largest group of bacteria in the gut, members of the phylum *Firmicutes*, ferment dietary fiber to short-chain fatty acids (SCFAs), such as butyrate, which are used as energy sources and are regulators of inflammation (Tremaroli and Bäckhed, 2012). In addition, *Firmicutes* species, together with members of phylum *Bacteroidetes*, are involved in the regulation of lipid and bile acid metabolism and in the maintenance of energy balance in the host (Pan et al., 2018). There is substantial evidence to support an increase in *Proteobacteria* (especially adherent invasive *E. coli*) in IBD patients (Knox et al., 2019). In our study, UC patients with active disease had significantly higher levels of *Proteobacteria* than UC patients in the remission and healthy control subjects, and this suggests that *Proteobacteria* abundance may be related to the severity of inflammation. Several other studies investigating the microbiota in UC patients using mucosal biopsy have also reached similar conclusions (Lucke et al., 2006; Chen et al., 2014; Ahmed et al., 2016; Ma et al., 2018).

At the genus level, we found that abundance levels of many bacteria were altered in UC patients, which was reflected in the LEfSe results. *Blautia* and *Lachnoclostridium* were significantly elevated in UC patients in remission, whereas *Rothia*, *Actinomyces*, *Pediococcus*, and *TM7x* could be considered biomarkers in patients with active disease. *Blautia* is an anaerobic bacterium with probiotic characteristics that promotes the production of SCFAs, prevents inflammation, and maintains intestinal homeostasis (Liu et al., 2021), and has been found to be significantly reduced in colorectal cancer patients (Chénard et al., 2020) and in animal models (Zhang et al., 2020). In addition, previous research has found that *Lachnoclostridium* in stool could serve as a biomarker for non-invasive diagnosis of colorectal adenoma and cancer (Liang et al., 2020). *Rothia dentocariosa* is a conditioned pathogen found mainly in the human oral cavity and is known to cause local periodontal infection, endocarditis, septicemia, peritoneal dialysis-related peritonitis, and pneumonia (Yeung et al., 2014). Our findings reveal the need to be vigilant in monitoring the pathogenicity of this commensal bacterium in patients with active UC and its potential to cause clinically significant infection. *TM7x* is the first obligate epibiont parasite discovered within the domain of Bacteria that requires other bacteria (*Actinomyces odontolyticus* strain XH001) as a host (Ibrahim et al., 2021). *Actinomyces* species are opportunistic pathogens mostly inhabiting the oral cavity and upper respiratory tract, and overgrowth of this genus could cause inflammation. Therefore, UC patients need to pay attention to oral hygiene and ensure timely treatment of oral diseases. The term ‘gum–gut axis’ was first proposed by Kevin and Ajay (Byrd and Gulati, 2021) to describe the relationship between the microbiota of the oral cavity and the gastrointestinal tract. Other studies have since reported the presence of many oral microbes in the intestinal tissues of IBD patients, such as *Aggregatibacter*, *Campylobacter*, *Enterobacteria*, *Fusobacterium*, *Gemella*, *Neisseria*, *Pasteurella*, *Peptostreptococcus*, and *Streptococcus* (Gevers et al., 2014; Yachida et al., 2019; Schmidt et al., 2019). We observed the colonization of distant microbial niches by oral microbes in UC patients with acute disease, which can complement the knowledge and understanding of the gum–gut axis.

Previous research has shown that, compared with healthy individuals, the gut microbiota of UC patients and *Bifidobacterium* species, and a lower abundance of *Faecalibacterium prausnitzii*, as well as a lower concentration of organic acids (Sartor and Wu, 2017; Knox et al., 2019). Sulfate-reducing bacteria, whose metabolites (e.g. hydrogen sulfide) can block the use of butyrate by colonocytes (Smith et al., 2005), have also been found to be increased in abundance in patients with IBD (Verma et al., 2010). Lloyd-Price et al. (Lloyd-Price et al., 2019) previously demonstrated a characteristic increase in facultative anaerobes at the expense of obligate anaerobes in the gut microbiota of UC patients, and these previous findings were also reflected in our results. Subsequent studies on the IBD microbiome should involve specific strains that can provide greater insight into correlated changes in the microbiome.

We also found that the relative abundances of *Sutterella*, *Haemophilus*, and *Klebsiella* species were significantly higher in UC patients with active disease than in those in remission. *Sutterella* is known to impair gut antibacterial immune function and increase susceptibility to gut disease (Moon et al., 2015). Enrichment of *Sutterella* leads to excessive secretion of IgA protease and, thus, a decreased IgA concentration in the intestinal mucosa, which facilitates pathobiont invasion of epithelial cells and intracellular survival (Kaakoush, 2020). Interestingly, the relative abundance of *Butyricicoccus*, a recognized pathogenic bacterium, was higher in UC patients in remission than in those with active disease. *Butyricicoccus* is known to prevent cytokine-induced epithelial integrity losses and promote the proliferation of beneficial flora (such as *Bifidobacterium* and *Lactobacillus*) (Eeckhaut et al., 2013; Devriese et al., 2017). In addition, we found no significant difference in *Faecalibacterium prausnitzii* abundance among the three groups, similar to the finding of Brigida et al. (Barberio et al., 2022), but in contrast to the vast majority of previous studies (Sokol et al., 2009; Machiels et al., 2014). This may be related to the ethnic and geographical differences of UC patients, and follow-up of a large number of Asian UC patients will be necessary to explore this issue.

To our knowledge, this is the first trial to report a positive correlation between *Roseburia* and *Lachnospira* in gut microbiota, especially in UC patients. Both *Roseburia* and *Lachnospira* are butyrate-producing genera, and they play an important role in the control of intestinal inflammatory processes (Wu et al., 2020; Nakamura et al., 2022).

As is widely known, microorganisms have an impact on human immunity, the degradation and absorption of nutrients, and even the metabolism of substances (Martin et al., 2010). To further assess the impact on the host of a change in gut microbiome, we employed PICRUSt2 analysis. Our results suggest that the levels of methane, lipopolysaccharide, geraniol, ansamycins, valine, leucine, and isoleucine, among others, are increased in UC patients with severe inflammation. This finding has only rarely been reported before and might play a certain anti-inflammatory role in the clinical course of disease in UC patients.

It should be noted that our study also had certain limitations. The focus of our research was on dysbiosis in the stool of UC patients, and the perturbation of the structure of the gut microbiota in the biopsy mucosa deserves further research. Furthermore, owing to its cross-sectional design, our study was unable to dynamically observe changes in the intestinal flora of the same UC patient during the recurrence and remission stages. Extensive effort is required to identify changes in the microbiota and their role in the pathogenesis of UC.

Overall, our study revealed that the bacterial community composition of stool differs between UC patients with active disease and those in remission. Our findings will certainly lay the foundation for further research on the function and signaling pathways of gut microbiota in UC. Further comprehensive investigations of microbiomics and metabonomics have great potential to expand our knowledge of the pathogenesis of IBD and to identify potential treatments.

## Data availability statement

The name of the repository is NCBI Sequence Read Archive (SRA) database, and accession number is PRJNA866870.

## Ethics statement

The studies involving human participants were reviewed and approved by the Medical Ethical Committee of the Beijing Friendship Hospital Affiliated to Capital Medical University. The patients/participants provided their written informed consent to participate in this study. Written informed consent was obtained from the individual(s) for the publication of any potentially identifiable images or data included in this article.

## Author contributions

Conceived and designed the experiments: SZ, HS, and PL. Collection of fecal specimens: SL and LF. Performed the experiments: SZ and MH. Analyzed the data: MH and SZ. Manuscript preparation: MH. Manuscript revisions: SZ, HS, and PL. All authors have approved the final version of the manuscript and agree to be accountable for all aspects of the work, ensuring that questions related to the accuracy or integrity of any part of the work are appropriately investigated and resolved.

## Funding

This work was funded by the National Natural Science Foundation of China (82070575); Beijing Municipal Administration of Hospitals’ Youth Programme (QML20190104, QML20180102); Beijing Nova Program (Z201100006820147); Beijing Municipal Science & Technology Commission (Z181100001718221).

## Acknowledgments

We thank Allwegene Company (Beijing) for technical assistance in sequencing.

## Conflict of interest

The authors declare that the research was conducted in the absence of any commercial or financial relationships that could be construed as a potential conflict of interest.

## Publisher’s note

All claims expressed in this article are solely those of the authors and do not necessarily represent those of their affiliated organizations, or those of the publisher, the editors and the reviewers. Any product that may be evaluated in this article, or claim that may be made by its manufacturer, is not guaranteed or endorsed by the publisher.

## Supplementary material

Supplementary Figure 1 The microbiota basic line of the subjects**. (A)** Sequence distribution map. The abscissa shows the sequence length gradient, and the ordinate the number of tags. **(B)** Venn diagram. Different colors represent different samples, and overlapping circles represent OTUs that occur in more than one sample.

Supplementary Figure 2 The microbiota basic analysis of the subjects. **(A)** Rarefaction curve. The abscissa shows the number of randomly selected sequencing data, and the ordinate the number of observed OTUs. **(B)** Shannon–Wiener curve. The abscissa shows the sequencing depth and the ordinate Shannon Diversity Index. **(C)** Species accumulation curves. The abscissa shows the sample size, and the ordinate the number of OTUs after sampling. **(D)** Rank abundance curve. The abscissa shows the OTU level, and the ordinate the relative percentage of sequence numbers in OTUs of this level, that is, the number of sequences belonging to this OTU divided by the total number of sequences. AUC, UC patients with active disease; RUC, UC patients in remission; and HC, healthy control subjects.

Supplementary Figure 3 Network interaction diagram. The size of the dot represents the abundance, the thickness of the line represents the correlation, the color of the dot represents the phylum it belongs to, and the red line represents a positive correlation.

Supplementary Figure 4 Bar plot of differentially enriched metabolic pathways at KEGG level 3. AUC, UC patients with active disease; RUC, UC patients in remission; and HC, healthy control subjects.
