# Supplementary figures and images for: Composition and diverse differences of intestinal microbiota in ulcerative colitis patients
Source: Front Cell Infect Microbiol. 2022 Aug 30;12:953962. doi: 10.3389/fcimb.2022.953962 (PMC9468541; doi:10.3389/fcimb.2022.953962)

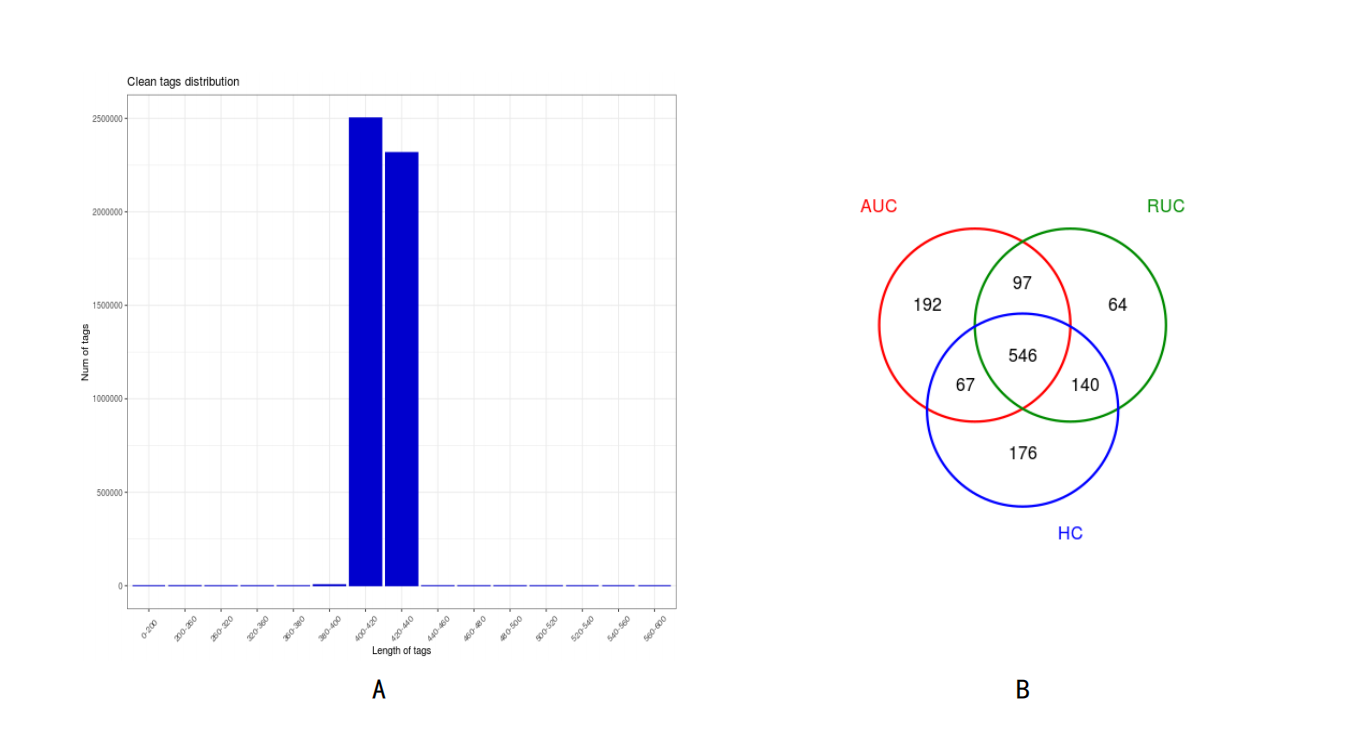

Supplement: Supplementary Figure 1 — The microbiota basic line of the subjects. (A) Sequence distribution map. The abscissa shows the sequence length gradient, and the ordinate the number of tags. (B) Venn diagram. Different colors represent different samples, and overlapping circles represent OTUs that occur in more than one sample. [file Image_1.tif]

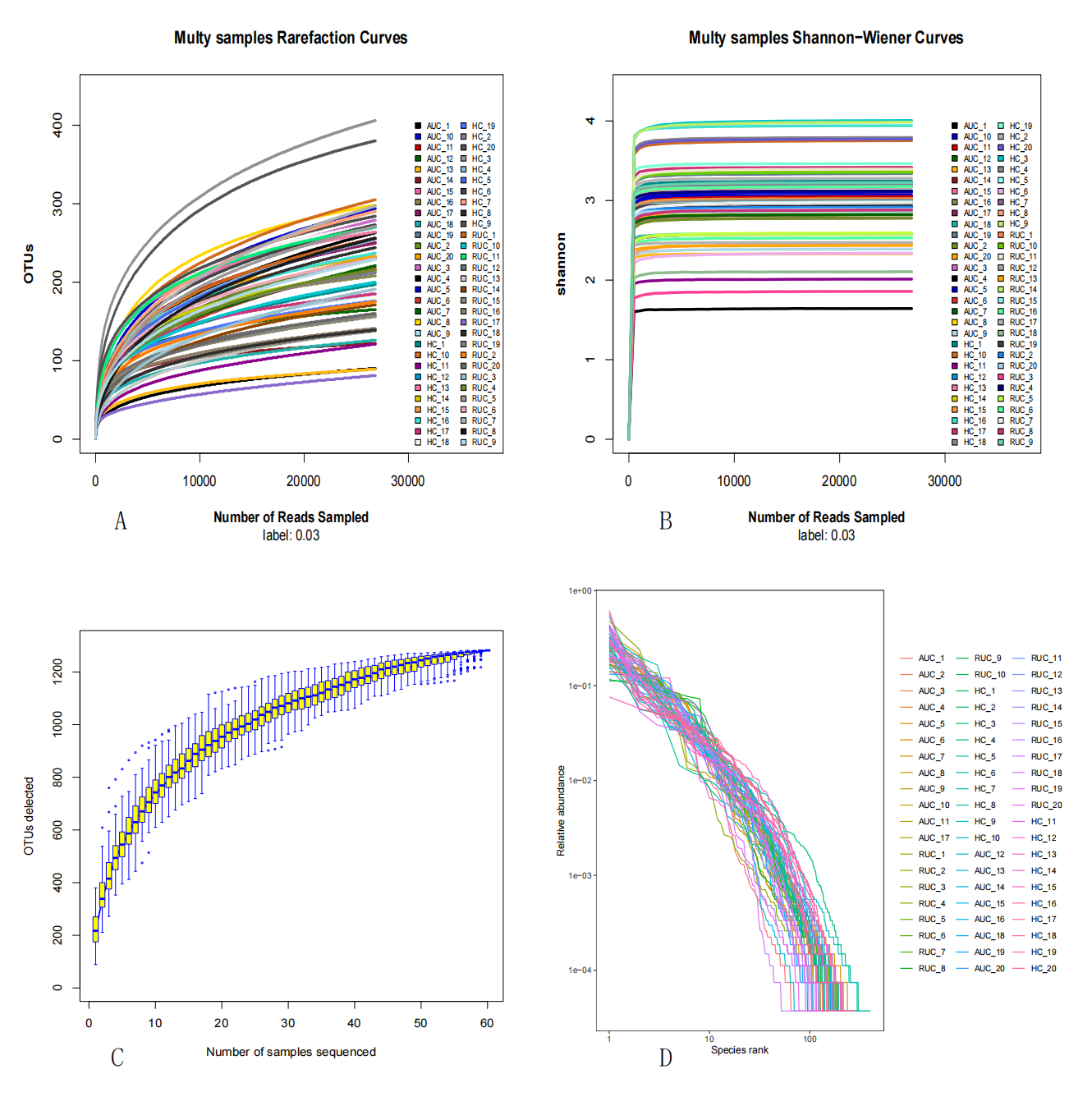

Supplement: Supplementary Figure 2 — The microbiota basic analysis of the subjects. (A) Rarefaction curve. The abscissa shows the number of randomly selected sequencing data, and the ordinate the number of observed OTUs. (B) Shannon–Wiener curve. The abscissa shows the sequencing depth and the ordinate Shannon Diversity Index. (C) Species accumulation curves. The abscissa shows the sample size, and the ordinate the number of OTUs after sampling. (D) Rank abundance curve. The abscissa shows the OTU level, and the ordinate the relative percentage of sequence numbers in OTUs of this level, that is, the number of sequences belonging to this OTU divided by the total number of sequences. AUC, UC patients with active disease; RUC, UC patients in remission; and HC, healthy control subjects. [file Image_2.tif]

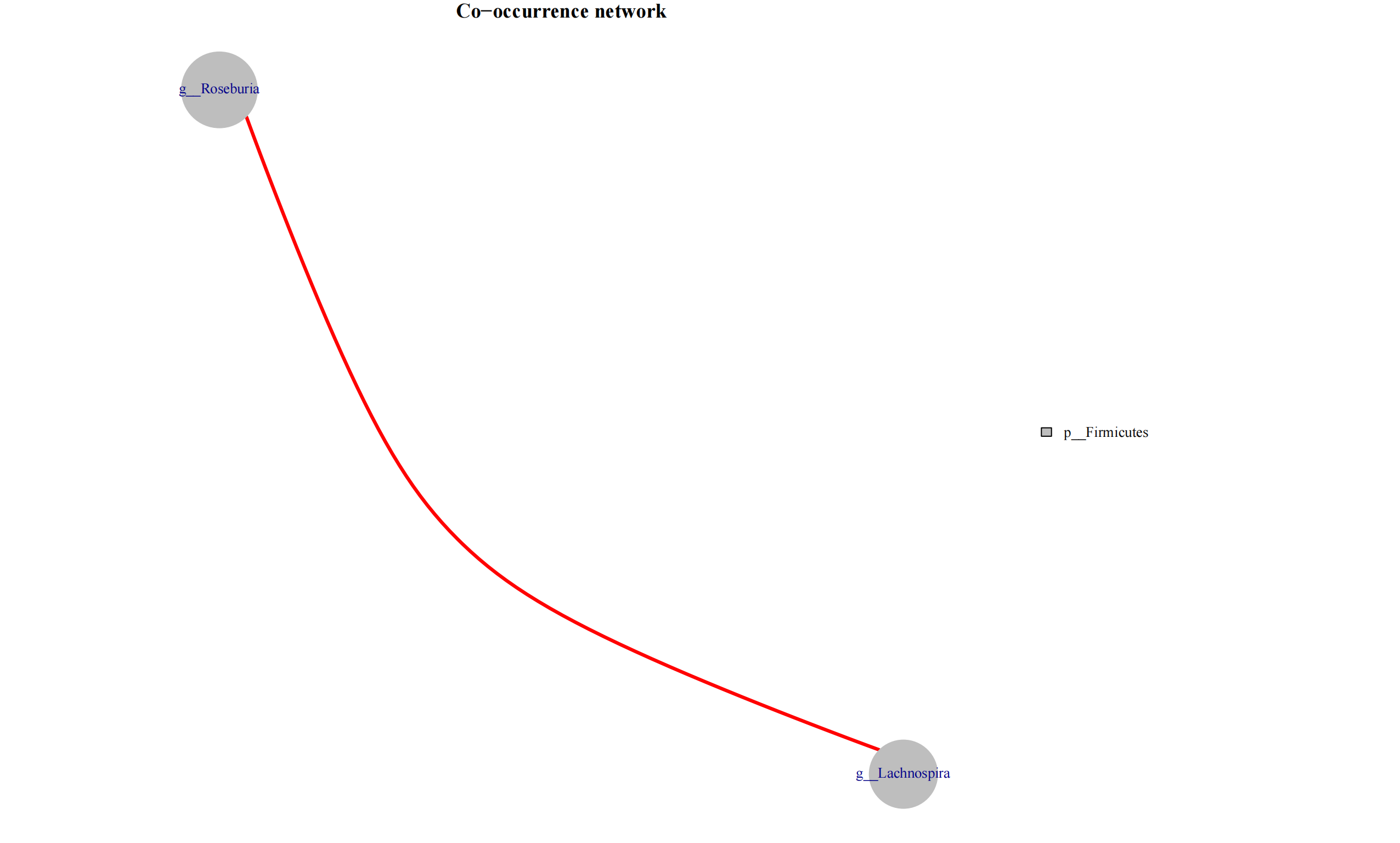

Supplement: Supplementary Figure 3 — Network interaction diagram. The size of the dot represents the abundance, the thickness of the line represents the correlation, the color of the dot represents the phylum it belongs to, and the red line represents a positive correlation. [file Image_3.tif]

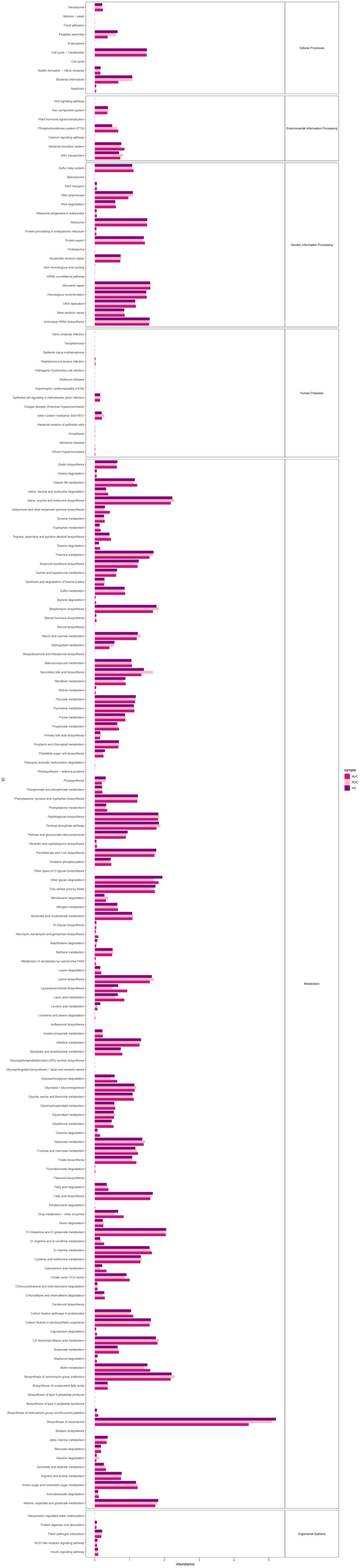

Supplement: Supplementary Figure 4 — Bar plot of differentially enriched metabolic pathways at KEGG level 3. AUC, UC patients with active disease; RUC, UC patients in remission; and HC, healthy control subjects. [file Image_4.jpeg]
